# New Frontiers in Measuring and Characterizing the HIV Reservoir

**DOI:** 10.3389/fmicb.2019.02878

**Published:** 2019-12-18

**Authors:** Shane D. Falcinelli, Cristina Ceriani, David M. Margolis, Nancie M. Archin

**Affiliations:** ^1^UNC HIV Cure Center, The University of North Carolina at Chapel Hill, Chapel Hill, NC, United States; ^2^Department of Microbiology and Immunology, The University of North Carolina at Chapel Hill, Chapel Hill, NC, United States; ^3^Department of Medicine, The University of North Carolina at Chapel Hill, Chapel Hill, NC, United States

**Keywords:** HIV, cure, reservoir, stability, replication-competent, defective provirus, QVOA, IPDA

## Abstract

A cure for HIV infection remains elusive due to the persistence of replication-competent HIV proviral DNA during suppressive antiretroviral therapy (ART). With the exception of rare elite or post-treatment controllers of viremia, withdrawal of ART invariably results in the rebound of viremia and progression of HIV disease. A thorough understanding of the reservoir is necessary to develop new strategies in order to reduce or eliminate the reservoir. However, there is significant heterogeneity in the sequence composition, genomic location, stability, and expression of the HIV reservoir both within and across individuals, and a majority of proviral sequences are replication-defective. These factors, and the low frequency of persistently infected cells in individuals on suppressive ART, make understanding the reservoir and its response to experimental reservoir reduction interventions challenging. Here, we review the characteristics of the HIV reservoir, state-of-the-art assays to measure and characterize the reservoir, and how these assays can be applied to accurately detect reductions in reservoir during efforts to develop a cure for HIV infection. In particular, we highlight recent advances in the development of direct measures of provirus, including intact proviral DNA assays and full-length HIV DNA sequencing with integration site analysis. We also focus on novel techniques to quantitate persistent and inducible HIV, including RNA sequencing and RNA/*gag* protein staining techniques, as well as modified viral outgrowth methods that seek to improve upon throughput, sensitivity and dynamic range.

## Introduction

An estimated 40 million people are living with HIV, and over 30 million have died because of it. The incidence of new HIV infections globally is approximately 2 million each year (GBD 2015 HIV Collaborators, 2016; WHO, 2019). While antiretroviral therapy (ART) can control viremia and improve health outcomes, durably ART-suppressed HIV seropositive individuals are still at higher risk for diseases related to immune dysfunction and inflammation compared to uninfected individuals (Hunt et al., 2016). Moreover, lifelong adherence to ART is required for optimal health outcomes and is associated with financial burden, drug interactions, and unknown long-term effects of treatment (Deeks et al., 2017; Devanathan et al., 2019). Further, the stigma associated with HIV disease contributes to negative health outcomes (Rueda et al., 2016). Therefore, a cure for HIV is of substantial public health interest.

However, eliminating HIV infection remains an elusive goal due to the viral reservoir—HIV proviral DNA in the host genome of resting CD4+ T lymphocytes and possibly other cells (Bukrinsky et al., 1991; Chun et al., 1997, 1998; Finzi et al., 1997; Wong et al., 1997b; Finzi et al., 1999; Ganor et al., 2019). For the purposes of this review, we define the viral reservoir as a cell that encodes integrated, replication-competent virus that can fuel viral rebound after treatment interruption, even after years of suppressive ART (Eisele and Siliciano, 2012; Margolis et al., 2016). When referring broadly to HIV DNA that is integrated but includes both replication-competent and replication-incompetent HIV, we use the term persistent HIV (Wang et al., 2018b). On suppressive ART, transcription of proviral DNA from persistently infected cells during immune activation or other normal cellular processes can result in viral gene expression and viral particle release (Shen and Siliciano, 2008; Pasternak et al., 2013). Whether these particles are able to establish new infections under suppressive ART is a historical area of debate (Shen and Siliciano, 2008; Lorenzo-Redondo et al., 2016; Margolis et al., 2016; Martinez-Picado and Deeks, 2016); however, the overwhelming majority of evidence supports the notion that active viral replication does not occur during suppressive therapy. Rather, (1) long-lived cells infected prior to ART-initiation and (2) expansion of infected cells through cell division during suppressive therapy are responsible for persistence of the reservoir on long-term suppressive ART (Maldarelli, 2015; Bui et al., 2017; Kearney et al., 2017; Anderson and Maldarelli, 2018; Mok et al., 2018; Wang et al., 2018a; Bozzi et al., 2019). Nonetheless, upon cessation of suppressive ART, there is a rebound of viremia from the reservoir on the timescale of weeks and in general, progression of HIV disease if ART is not resumed (Chun et al., 1999; Hamlyn et al., 2012).

Research efforts over the past 30 years have sought to understand the reservoir in order to facilitate the development of interventions that could eradicate the reservoir (a cure) or enable HIV seropositive individuals to maintain suppressed viremia in the absence of ART (a functional cure) (Deeks et al., 2016). A central strategy for eradication (cure) is the use of latency reversal agents (LRAs) to induce proviral expression in conjunction with a vaccine, antibody, and/or cellular immunotherapy in order facilitate immune clearance of virally infected cells (Margolis et al., 2016). With regard to a functional cure, major strategies include the use of long-acting broadly neutralizing antibodies (passive immunotherapy), vaccination (active immunotherapy), modification of host cells to prevent viral replication (gene therapy), or permanent silencing of provirus (“block and lock”) (Cillo and Mellors, 2016; Kessing et al., 2017; Elsheikh et al., 2019).

Common to all of these approaches for a cure or functional cure is a need for careful characterization and measurement of the viral reservoir. In recent years, advances in technology and the HIV cure field have resulted in the development of an arsenal of assays to characterize and measure the HIV reservoir. In this review, we first provide an overview of the characteristics of HIV reservoir—including the cell types thought to harbor the reservoir, the spectrum of viral expression within the reservoir, and the diversity of proviruses within the reservoir. Next, we review standard as well as emerging assays for measuring the reservoir. Throughout, we discuss ongoing and future applications of these assays to (1) improve understanding of the stability of the HIV reservoir and (2) accurately and reliably detect a reduction in the frequency of the HIV reservoir in cure research.

## The Hiv Reservoir: Cell Types That Harbor the Reservoir

Here, we define the HIV reservoir as any cell that can produce replication-competent virus despite years of suppressive ART (Eisele and Siliciano, 2012; Margolis et al., 2016). To date, in humans, resting CD4+ T lymphocytes represent the largest and most well-characterized viral reservoir. However, there is mounting evidence from both animal models and human donors that other cell types may contribute to the total HIV reservoir. To provide context for our review of assays to measure the reservoir, we provide a brief overview of the cell types that may compose the reservoir.

### CD4 T Cells

Resting CD4 T cells, defined as CD3+CD4+CD25-CD69- HLADR-, were the first cells to be identified as a reservoir for HIV (Chun et al., 1997, 1998; Finzi et al., 1997, 1999; Wong et al., 1997b). At the time, it was generally not believed that HIV exhibited a latent stage in its lifecycle, and so the use of this resting cell population allowed the demonstration of a definable pool of cells that lacked HIV expression when isolated, but could be induced (activated) *ex vivo* to express replication-competent HIV — hence a definitive proof of the presence of true virologic latency.

**FIGURE 1:**
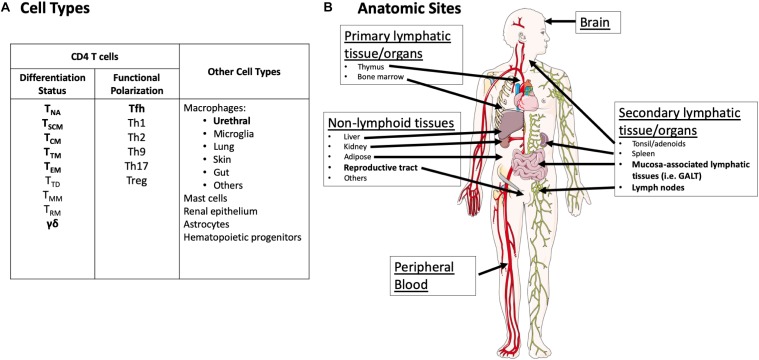
Overview of cell types and anatomic sites reported to harbor the latent reservoir. **(A)** Cell types thought to harbor the HIV reservoir. Cell types with demonstrated recovery of replication-competent virus (defined as propagating virus in an outgrowth assay) in humans following years of suppressive ART are in bold. Cell types in regular font represent cells where HIV nucleic acid has been detected by PCR and/or sequencing either in humans or animal models but recovery of replication-competent virus in humans after years of suppressive ART has not been demonstrated. It is important to note that for many cell types, there has been very sparse sampling for replication-competent virus. NA, naïve; SCM, stem cell memory; CM, central memory; TM, transitional memory; EM, effector memory; TD, terminally differentiated; MM, migratory memory; RM, resident memory. **(B)** Anatomic sites with demonstrated recovery of replication-competent virus in humans following years of suppressive ART are highlighted in bold. Potential replication-competent anatomic reservoir sites are in regular font. These sites represent tissues/organs where HIV nucleic acid has been detected either in humans or animal models but recovery of replication-competent virus in humans after years of suppressive ART has not been demonstrated. It is important to note that for many tissue types, there has been very sparse sampling for replication-competent virus. Images were derived and modified from Servier Medical Arts under a Creative Commons Attribution 3.0 Unported License.

This reservoir in resting CD4 T cells is known to be very stable with a long half-life (∼44.5 months) (Siliciano et al., 2003; Crooks et al., 2015). Therefore, to date, these cells represent the most formidable barrier to cure because of their frequency and slow decay rate. Within resting CD4 T cells, the HIV reservoir is most frequently detected in the central memory compartment (Finzi et al., 1997; Soriano-Sarabia et al., 2014). Of note, some studies using total CD4 T cells have detected higher frequencies of persistent HIV in the effector memory subset (Hiener et al., 2017), whereas others describe the highest frequency of persistent HIV in the central memory compartment (Chomont et al., 2009). In addition, replication-competent HIV has been recovered from naïve T cells and transitional memory T cells (Chomont et al., 2009; Soriano-Sarabia et al., 2014; Zerbato et al., 2019). HIV reservoirs have also been detected in gamma/delta T cells (Soriano-Sarabia et al., 2015) (Figure 1). The half-life of the reservoir in each of these cell compartments is little studied, and is complicated by the natural differentiation of these immune cells across compartments (i.e., central memory to effector memory). Furthermore, in the case of gamma/delta T cells, their low frequency and dual function as reservoirs and immune effectors complicates efforts to understand their contribution as a stable source of HIV under ART (Soriano-Sarabia et al., 2015; Garrido et al., 2018). Finally, long-lived CD4+ T memory stem cells may also contribute significantly to the viral reservoir in some individuals (Buzon et al., 2014).

Non-resting CD4 T cells that express one or more markers associated with activation (CD25, CD69, and/or HLA-DR) may also contain persistent, replication-competent HIV; however, the stability of persistent HIV within these cells remains to be proven. HIV DNA is enriched in non-resting CD4 T cells (Chun et al., 2005) and markers of immune activation and dysfunction are moderately correlated with DNA and RNA markers of viral persistence in some studies (Chomont et al., 2009; Hatano et al., 2013; Cockerham et al., 2014). A recent study demonstrated the recovery of identical *gag-pol* sequences from HLA-DR expressing-CD4 T cells over multiple time points in durably suppressed participants (Lee et al., 2019). This underscores the importance of interrogating non-resting CD4 T cells for HIV during suppressive therapy. Whether virus found in non-resting T cells originated from resting cells that acquired activation markers, or if some of these cells later return to the resting state is not well understood. As most activated T cells die in the contraction phase of the immune response (Alderson et al., 1995; Swain et al., 1996; Green et al., 2003; Marrack et al., 2010; McKinstry et al., 2010), activated T cells likely do not represent a stable reservoir. However, it is unclear if HLA-DR, CD25, and/or CD69 expression are always indicators of fully activated T cells that are destined to die via immune contraction. In addition, CD25 and/or HLA-DR expressing cells likely represent a heterogenous population of T regulatory cells and activated T cells (Simonetta and Bourgeois, 2013; Corneau et al., 2017). Thus, further evaluation of persistent HIV within these non-resting cells is warranted.

Functional polarization of CD4 T cells may also influence reservoir formation and stability. Within the B cell follicle and in the periphery, T follicular helper (T_fh_) cells also represent an important cell type that harbors replication-competent HIV (Pallikkuth et al., 2015; Banga et al., 2016). Given the central role of T_fh_ cells in HIV replication during active infection, the reservoir may be highly enriched within tissue T_fh_ cells (Banga et al., 2016). In addition, HIV DNA is enriched in CD4 T cells with Th1/17 and Treg polarizations (Tran et al., 2008; Kulpa and Chomont, 2015; Sun et al., 2015; Dunay et al., 2017).

Studies of viremia in individuals on suppressive therapy (viral “blips” or residual viremia) demonstrate that the residual viremia is not always representative of the proviruses that compose the latent reservoir in CD4 T cells from peripheral blood (Bailey et al., 2006; Sahu et al., 2009; Anderson et al., 2011). However, this does not exclude the possibility that a small pool of peripheral blood CD4 T cells is responsible for residual viremia. Another possibility is that this residual viremia may be derived from CD4 T cells in tissues, or other cell types in tissues.

### Other Cell Types

Until recently no study has demonstrated recovery, following years of suppressive therapy, of replication-competent virus from any other purified human cell type other than CD4+ T cells. However, a recent study demonstrated recovery of replication-competent HIV from macrophages harvested from urethral tissue of durably ART-suppressed donors (Ganor et al., 2019). In addition to urethral macrophages, there are several lines of reasoning and evidence that underscore the need to evaluate other cell types for HIV reservoir.

Cells from non-lymphoid lineages can be infected and produce HIV virions during active infection. However, the role and stability of these cells under suppressive ART is highly debated. Bone-marrow derived macrophages, as well as yolk-sac derived microglia and other tissue-resident macrophages may also represent potential HIV reservoirs (Gomez Perdiguero et al., 2015; Gama et al., 2018; Wong et al., 2019). Importantly, there is an increasing appreciation for the ability of tissue resident macrophages to self-renew and potentially serve as a long-lived reservoir of replication-competent virus (Gama et al., 2018; Roszer, 2018; Wong et al., 2019). In addition, neuroepithelial progenitor-derived astrocytes also harbor HIV provirus, though the replication competency and potential for reactivation of this virus *in vivo* is poorly understood (Al-Harti et al., 2018; Barat et al., 2018). HIV can infect multipotent hematopoietic CD34+ progenitor cells; however, in general during stable therapy detection of HIV DNA in this population is rare and no recovery of replication-competent virus has been demonstrated (Carter et al., 2010; Durand et al., 2012; Josefsson et al., 2012; Sebastian et al., 2017; Zaikos et al., 2018). Similarly, HIV DNA has been detected rarely in monocytes from durably suppressed individuals; however, monocytes have a half-life of days *in vivo* and do not represent a stable reservoir. Furthermore, purification of monocytes is complicated by their tendency to stick to CD4 T cells (Burel et al., 2019; Massanella et al., 2019). Infectious virus was recovered from placenta-associated mast cells from women on suppressive ART; however, the period of ART was on a timescale of months rather than years and suppression was defined as <400 copies HIV RNA/mL of plasma (Sundstrom et al., 2007). In all of these cases, however, no longitudinal study of the viral sequences that would appear to be replication-competent, or the recovery of virus from these populations over time, has been demonstrated in any of these cell types.

While there is a substantial body of evidence supporting the presence of HIV DNA or RNA in these cell populations, there has been no detection of replication-competent HIV that is directly attributable to a non-lymphoid lineage in ART-suppressed human donors, except for urethral macrophages (Ganor et al., 2019; Wong et al., 2019). Nevertheless, the finding of persistent HIV in urethral macrophages despite long-term ART, demonstration of rebound viremia in humanized myeloid-only mice, and recovery of replication-competent SIV from brain macrophages of ART-suppressed macaques underscores the need to further evaluate non-lymphoid lineage cells as potential long-lived reservoirs of replication-competent HIV in humans (Honeycutt et al., 2017; Gama et al., 2018; Ganor et al., 2019).

## The Hiv Reservoir: Anatomic Sites of the Reservoir

The majority of studies of the HIV reservoir under suppressive ART have been in peripheral blood CD4 T cells. While these studies have yielded key insights into the HIV reservoir, it is critical to acknowledge that circulating CD4 T cells represent less than 2% of total body CD4 T cells (Westermann and Pabst, 1992; Di Mascio et al., 2009). There is an increasing appreciation for the microenvironment of tissue in the study of the reservoir of persistent HIV infection, and in the evaluation of cure or functional cure interventions (Figure 1) (Barton et al., 2016; Churchill et al., 2016; Wong and Yukl, 2016). This tissue context may include factors such as viral sequence compartmentalization, variation in viral expression levels, differential ART penetration, and availability of immune effector mechanisms to clear infected cells expressing viral RNA or protein (Fletcher et al., 2014; Deeks et al., 2016; Margolis et al., 2016; Baxter et al., 2018; van Zyl et al., 2018; Miller et al., 2019; Pardons et al., 2019).

Detailed HIV reservoir measurements of different tissues are reviewed elsewhere (Yukl and Wong, 2015; Barton et al., 2016; Churchill et al., 2016; Wong and Yukl, 2016). For the purposes of this review, we consider both defined and potential anatomic reservoir sites (Figure 1) in the context of current and emerging reservoir assays. Defined anatomic replication-competent HIV reservoir sites in humans who have been treated with long-term (years) of fully suppressive ART include CD4 T cells from peripheral blood and some secondary lymphoid tissues (Siliciano et al., 2003; Crooks et al., 2015; Sanyal et al., 2018), and recently the reproductive tract in the form of urethral macrophages (Ganor et al., 2019). It is critical to note that because of limited access to human tissue, there is sparse sampling of many tissue types (i.e., spleen) that undoubtedly harbor CD4 T cells with replication-competent HIV (Figure 1).

However, there are many long-lived cell types, such as other tissue resident macrophages in other sites (e.g., microglia), astrocytes, hematopoietic progenitor cells, among others, in which HIV DNA or RNA has been detected but replication-competent HIV has not (Carter et al., 2010; Canaud et al., 2014; Yukl and Wong, 2015; Al-Harti et al., 2018; Ko et al., 2019; Wong et al., 2019) (Figure 1). Therefore, we consider tissues with these cell types as potential reservoir sites. These cell types are found throughout the body in a variety of different tissues. Again, it is important to note that the absence of recovery of replication-competent virus in these tissues does not mean that reservoir does not exist in these locations. Limited access to human tissues, and the technical complexities associated with viral outgrowth assays in these samples limits the sensitivity of studies performed to date (Churchill et al., 2016; Deeks et al., 2016, 2017; Ganor et al., 2019). Understanding the composition and frequency of the replication-competent reservoir across different tissues is a critical issue for future cure research, as cure interventions must be assessed by their effect on the viral reservoir, wherever it is located.

## The Hiv Reservoir: Spectrum of Viral Expression, Diversity, and Replication Competency

HIV proviral expression and diversity varies greatly across cells, tissues, and individuals. Cells with proviruses in their genomic DNA can increase in frequency (through homeostatic or antigen-driven proliferation) or decrease in frequency (through activation-induced cell death, viral cytolysis, or immune clearance) (Chomont et al., 2009; Wang et al., 2018a). Importantly, in HIV+ individuals on stable, suppressive ART, the net effect of the above processes universally result in a very slow decay of the replication-competent HIV reservoir in resting CD4 T cells (Siliciano et al., 2003; Crooks et al., 2015).

Critically, the exact threshold of viral expression sufficient for viral cytolysis or immune recognition and clearance is not well defined. CD4 T cells expressing HIV RNA and sometimes viral proteins are often detectable in ART-suppressed individuals, in the absence of any latency reversal intervention (Baxter et al., 2018). These cells, which represent a portion of provirus that is competent for viral transcription and translation, may reach a threshold of viral expression that renders them susceptible to either immune clearance or cell death. Alternatively these cells expressing HIV RNA or protein may return to a more quiescent state and contribute to the maintenance of the HIV reservoir (Barton et al., 2016; Margolis et al., 2016). Careful definition of how much viral RNA and/or protein antigen is necessary to render an infected cell amenable to immune clearance or apoptosis is needed to inform cure strategies.

In addition to the wide spectrum of viral expression, the HIV reservoir also contains a large diversity of proviral sequences, reviewed in van Zyl et al. (2018). This diversity is primarily attributed to the absence of a proofreading mechanism in the HIV reverse transcriptase enzyme, as well as high levels of viremia and recombination (van Zyl et al., 2018). Other factors that contribute to this diversity include: host immune response (APOBEC enzyme hypermutations) (Bruner et al., 2016), length of infection (accumulation of mutations and depletion of CD4 T cell targets) (Arrildt et al., 2015; Zhou et al., 2016), viral compartmentalization (Yukl and Wong, 2015; van Zyl et al., 2018; Miller et al., 2019), and selection for viral quasispecies that are able to evade killing by host immune mechanisms (Phillips et al., 1991; Wei et al., 2003; Kearney et al., 2009; van Zyl et al., 2018).

Associated with this diversity is a significant frequency of replication-defective proviruses, as well as clonally expanded sequences that may or may not be replication-competent (Bruner et al., 2016; Bui et al., 2017; Wiegand et al., 2017). Critically, most estimates posit that over 90% of proviruses are replication-defective. This, combined with the low frequency (0.01–100 per 1,000,000 CD4 T cells) of replication-competent proviruses (Siliciano et al., 2003; Crooks et al., 2015; Bruner et al., 2019) makes measurement of the replication-competent HIV reservoir extremely difficult. Given the diversity of persistent HIV and large number of defective proviruses, it is critical to consider what each reservoir assay measures in the context of the scientific or clinical question being asked, and whether it overestimates or underestimates the true frequency of the replication-competent HIV reservoir.

## Measuring the Hiv Reservoir

To provide a framework for our review of HIV reservoir assays, we have organized our discussion into assays that (1) directly measure proviral genomes (i.e., DNA PCR and DNA sequencing-based assays) and (2) assays that measure basal or experimentally inducible persistent HIV expression (i.e., RNA, viral proteins, or propagating viral infection). The best use of each assay is dependent on the aims of the scientific study and the type(s) of samples available (Table 1).

**TABLE 1:** Overview of reservoir assays.

**Assay target**	**Assay**	**Sensitivity for HIV**	**Specificity for HIV**	**Specificity for intact provirus**	**Specificity for integrated provirus**	**Cost**	**Cell number requirement**	**Assay time requirement**	**Over/under estimation of replication- competent reservoir**	**Examples**
**DNA**	**qPCR/ddPCR (one region)**	Yes	Yes^∗∗^	No	No	Low	Low	Low	Over	Malnati et al., 2008; Strain et al., 2013; Bosman et al., 2015
	**Integrated DNA Assay**	Yes	Yes	No	Yes	Medium	Low	Medium	Over	Reviewed in Liszewski et al., 2009
	**IPDA**	Yes	Yes^∗∗^	Partial	No	Low	Low	Medium	Over	Bruner et al., 2019
	**Q4PCR**	Yes	Yes	Yes^∗^	No	Medium	Low	High	Over	Gaebler et al., 2019
	**SGS or NGS (one region)**	Yes	Yes	No	No	Medium	Low	High	Over	Reviewed in Wang and Palmer, 2018
	**Near-full length genome sequencing**	Yes	Yes	Yes^∗^	No	High	Medium	High	Over	Ho et al., 2013; Bruner et al., 2016; Hiener et al., 2017; Lee et al., 2017
	**Integration site + full length genome sequencing**	Yes	Yes	Yes^∗^	Yes	High	Medium	High	Over	Einkauf et al., 2019; Patro et al., 2019
**RNA**	**Bulk cell PCR**	Yes	Yes	No	N/A	Low	Low	Low	Over	Archin et al., 2012; Shan et al., 2013
	**Single cell PCR**	Yes	Yes	No	N/A	Low	Low	High	Over	Procopio et al., 2015; Yucha et al., 2017
	**Single copy assay (SCA)**	Yes	Yes	No	N/A	Medium	N/A	Medium	Over	Reviewed in Wang and Palmer, 2018
	**SGS or NGS**	Yes	Yes	No	N/A	Medium	Medium	High	Over	Wiegand et al., 2017; Das et al., 2018
	***In situ* hybridization/RNA flow**	Yes	Yes	No	N/A	Medium	Medium	Medium	Over	Zhang et al., 2018; Porichis et al., 2014; Martrus et al., 2016; Baxter et al., 2017; Grau-Exposito et al., 2017
**p24**	**FAST/Digital Microscopy**	Yes	Partial^∗∗∗^	Partial	N/A	High	Medium	High	Over	DeMaster et al., 2015
	**Flow Cytometry**	Yes	Partial^∗∗∗^	Partial	N/A	Medium	Medium	Medium	Over	Baxter et al., 2016; Martrus et al., 2016; Baxter et al., 2017; Pardons et al., 2019
	**ELISA (Digital and Standard)**	Yes	Partial^∗∗∗^	Partial	N/A	Medium	Medium	Medium	Over	Wu et al., 2017
	**Mass Cytometry**	Yes	Partial^∗∗∗^	Partial	N/A	Medium	Medium	Medium	Over	Cavrois et al., 2017
**Outgrowth**	**QVOA**	Yes	Yes	Yes	N/A	High	High	High	Under	Siliciano and Siliciano, 2005; Archin et al., 2017

*^∗^Sequencing assays slightly overestimate the replication-competent reservoir. ^∗∗^ddPCR-based assays are associated with low false positive rates. ^∗∗∗^p24-antibody based assays have false positive rates.*

## Measuring the Hiv Reservoir: Direct Measurement of Viral Genomes

### DNA PCR Assays

One of the first, and to date most widely used methods to estimate the frequency of persistent HIV is the detection of HIV DNA by quantitative PCR (qPCR). Of note, these assays all measure proviruses directly, but do not provide any information about their inducibility. An economical and rapid method, DNA PCR assays have been widely applied in different cell types and tissues (Malnati et al., 2008; Chomont et al., 2009; Yukl et al., 2013). However, it is often difficult to compare DNA PCR measurements across different assays (assay performance characteristics and cell normalization methods), individuals (proviral sequence heterogeneity) and tissues (differential extraction efficiencies and levels of PCR inhibitors across tissues) (Henrich et al., 2017). Specific DNA PCR assays are too numerous to discuss here, but primarily target conserved regions of *gag*, the LTR, or *pol*.

Digital droplet PCR (ddPCR) assays for HIV DNA have also been developed, and in general relative to qPCR have greater precision and tolerance for primer/probe mismatches, which are common because of the sequence heterogeneity of proviral DNA (Strain et al., 2013; Rutsaert et al., 2018). One limitation of HIV ddPCR assays, however, is the presence of false positives in no template control (NTC) wells and in DNA samples from HIV seronegative participants. This limitation, discussed in detail elsewhere (Strain et al., 2013; Kiselinova et al., 2014; Bosman et al., 2015; Rutsaert et al., 2018), is problematic for participants with very low reservoir frequencies. In contrast, false positives in qPCR assays occur much less frequently, and false positives can be excluded by sequencing, which is not possible on current ddPCR platforms (Strain et al., 2013; Bosman et al., 2015). Thus, for discriminating the absolute presence or absence of HIV DNA, qPCR-based approaches are preferred.

qPCR and ddPCR DNA assays overestimate the frequency of replication-competent reservoir primarily because there are many mutations with a replication-defective phenotype that occur outside of the viral region assayed, including APOBEC hypermutations, insertions, deletions, and packaging signal defects (Bruner et al., 2015, 2016). In addition, because most DNA PCR primers are designed inside the HIV genome region, these assays also detect linear unintegrated DNA and episomal forms of HIV DNA such 2-long terminal repeat (2-LTR) circles, though these unintegrated forms are significantly less frequent, and their stability in durably ART-suppressed individuals is debated (Eriksson et al., 2013; Bruner et al., 2015; Martinez-Picado et al., 2018). To overcome the problem of unintegrated forms of HIV DNA, assays for integrated HIV DNA were developed, taking advantage of the high frequency (∼11%) of Alu short interspersed elements within the human genome as a host target to guarantee the exclusion of unintegrated forms of HIV (Deininger, 2011). One primer targets Alu elements while the other primer targets *gag*, followed by a nested PCR for the HIV LTR, enabling the detection of integrated provirus (O’Doherty et al., 2002; Liszewski et al., 2009). However, these assays are subject to variable efficiency depending on the proximity of human Alu sequence and the HIV genome that requires correction, and often require a high number of replicates for accurate frequency estimation (Liszewski et al., 2009; Graf and O’Doherty, 2013). Other methods to assay for integrated HIV DNA, including gel fractionation, inverse PCR, and linker ligation PCR, are reviewed in Liszewski et al. (2009).

In most PCR assays, the use of primers and probes targeting only one highly conserved proviral region detect HIV genomes that are defective or mutated in regions external of the assay amplicon (Eriksson et al., 2013; Bruner et al., 2016). Recent efforts have developed assays to simultaneously detect several conserved regions of the HIV genome. Bruner et al. (2019) developed the Intact Proviral DNA Assay (IPDA) that, based on ddPCR multiplex technology, simultaneously interrogate the presence of an intact packaging signal (PS) and the Rev-responsive element (RRE) within *env*. This innovative approach enables estimates of intact proviral DNA with as few as 5 million CD4 T cells. Moreover, the IPDA does not depend on long-distance PCR, the inefficiency of which might limit the ability of near full-length DNA sequencing approaches to accurately estimate the frequency of intact provirus. However, because the IPDA detects only a small sub-genomic region (2% of the HIV genome), there is still a risk of overestimation of intact proviral DNA. The IPDA may also quantify possible non-inducible provirus that may permanently remain latent for the lifetime of an individual. Bruner et al. (2019), in a bioinformatics analysis of several hundred proviral sequences, report that of the proviruses that would be amplified by the IPDA, approximately 70% are intact. To what extent this percentage varies from participant to participant, however, was not studied as it was not possible to perform sequencing following ddPCR in this study.

This knowledge gap was recently addressed by Gaebler et al. (2019), using a combined quadruplex qPCR and next-generation sequencing approach, termed Q4PCR. Q4PCR employs probes for PS, *env* (RRE), *pol* and *gag* that are highly conserved in intact proviruses. Q4PCR employs the same primer/probe set for *env* (RRE) as in the published version of the IPDA as well as the patent application (Siliciano, 2016; Bruner et al., 2019; Gaebler et al., 2019). Compared to the published version of the IPDA (Bruner et al., 2019), the primers for the PS region are different in Q4PCR (Gaebler et al., 2019); however, the probe is nearly identical. The PS primers and probe used in Q4PCR are identical to the sequences in the patent application for the IPDA (Siliciano, 2016).

Gaebler et al. (2019), in a separate bioinformatics analysis of >1000 near-full length sequences found that approximately 60% of proviruses detected by the PS and *env* primer/probe combination were intact. However, in sequence analysis of six individuals, they found that no amplification occurred for PS and *env* probes in 2/6 individuals. This variability was attributed to sequence polymorphisms in proviral genomes across individuals. While a potential issue, it appears to occur relatively infrequently (Bruner et al., 2019) and is readily corrected with alternative primers. In four individuals studied by Gaebler et al. (2019), the percentage of proviruses called intact by the IPDA probes that were truly intact by near-full-length sequencing varied from 9.1 to 96%. Gaebler et al. (2019) findings highlight a potential limitation of the IPDA: the percentage of proviruses that are correctly called intact by the IPDA may vary widely between individuals. This may be less troublesome if the assay is used to measure the effect of an intervention in an individual, but problematic if the assay is used to categorize different individuals. Further evaluation of more individuals with head-to-head comparisons of the Q4PCR method (modified to include the most current version of the IPDA PS primer sequences) and the IPDA is warranted. Moreover, development of intact proviral DNA assays that are optimized for all HIV subtypes will be necessary (Bruner et al., 2019). Nonetheless, the IPDA represents an attractive addition to the reservoir assay toolkit, as it is currently the most accurate high-throughput DNA assay for estimating the size of the replication-competent reservoir (Bruner et al., 2019).

### HIV DNA Sequencing

Sequencing of HIV proviral genomes enables measurements of reservoir frequency, diversity, and evolution. While single genome/proviral sequencing (SGS/SPS) assays provide important information about proviral diversity, they overestimate the replication competent reservoir because they are based on sequencing of subgenomic regions and miss proviruses with deletions or mutations in the primer binding sites (Wang and Palmer, 2018). To reduce the former limitation, several near full-length HIV sequencing assays were developed (Ho et al., 2013; Bruner et al., 2016; Hiener et al., 2017; Lee et al., 2017) and are reviewed in Wang and Palmer (2018) and Wang et al. (2018b). Although laborious and expensive, they provide an assessment of persistent HIV diversity across different cell subsets and tissues, and with longitudinal sampling, could provide insight into the dynamics of reservoir frequency and diversity over time (Hiener et al., 2017; Lee et al., 2017). Limitations of these near-full-length sequencing assays include poor coverage of the viral LTRs and slight overestimation of replication-competent reservoir frequency (Wang and Palmer, 2018; Einkauf et al., 2019). In addition, application of these technologies to a higher number of individuals and time points is necessary to make generalizable findings about the sequence composition of the HIV reservoir (Wang et al., 2018b).

Sequencing of HIV integration sites has also provided critical insights into the HIV reservoir. Initial integration site mapping efforts underscored the importance of clonal expansion in HIV reservoir maintenance (Maldarelli et al., 2014; Wagner et al., 2014). Simultaneous detection of near-full length provirus as well as its integration site could improve knowledge of the reservoir and inform cure strategies. Two research groups are developing integrated full length HIV sequencing assays capable of linking HIV genome sequences and their integration site. Both the methods use a multiple displacement amplification (MDA) combined with SGS (Full length integrated proviral single genome sequencing, FLIP-SGS) (Patro et al., 2019) or followed by NGS (matched integration site and proviral sequencing, MIP-Seq) (Einkauf et al., 2019). These methods may yield important insights into reservoir dynamics and formation, as well as reservoir maintenance through clonal expansion. Early studies suggest that in participants on durable therapy, intact proviruses, relative to defective proviruses, are enriched in non-genic regions of the genome (Einkauf et al., 2019).

## Measuring the Hiv Reservoir: Assessment of Basal or Inducible Reservoir Expression

Cell-associated HIV RNA is readily detectable at baseline, even in resting CD4 T cells (Archin et al., 2012; Archin et al., 2017). This suggests that at least in some cells, HIV is not completely quiescent. Thus, measurements of persistent HIV can be made on (1) unstimulated cells or (2) cells stimulated with T cell activating agents (the inducible reservoir). It is important to note for assays that measure the inducible reservoir, whether at the level of RNA, protein, or viral outgrowth, not all replication-competent proviruses are induced by a single round of T cell activation (Ho et al., 2013).

### HIV RNA PCR

While proviruses that are transcription-competent are more likely to be replication-competent than any given sample of proviral DNA, there are still many defective proviruses that can be expressed at the RNA level (Bruner et al., 2016; Imamichi et al., 2016; Pollack et al., 2017; Wiegand et al., 2017). That said, baseline ca-RNA expression (and bulk HIV DNA) is predictive of time to viral rebound upon therapy cessation and may serve as a biomarker to guide future analytical treatment interruption studies (Pasternak and Berkhout, 2018).

Unlike proviral DNA where there is (generally) one integrated copy per infected cell, many copies of RNA can be present in a single cell, which makes reservoir frequency measurements using RNA more challenging. Bulk qPCR or ddPCR assays of HIV RNA can provide a sensitive and low cost estimation of HIV transcription. Of note, for one reported HIV-RNA assay, semi-nested qPCR appeared to have greater quantitative linearity, accuracy, and sensitivity relative to ddPCR (Kiselinova et al., 2014). However, these assays of bulk RNA cannot determine the frequency of HIV RNA positive cells, or the phenotype of the cell(s) expressing HIV RNA. Furthermore, similar issues discussed above in the DNA PCR section for comparisons across assays, individuals, and tissues apply to RNA as well. Targets of cell-associated RNA assays include assays for unspliced *gag* (Archin et al., 2012), polyadenylated transcripts (Shan et al., 2013), among others (Fischer et al., 2002; Pasternak et al., 2013; Pasternak and Berkhout, 2018). In addition, Yukl et al. (2018) recently published a ddPCR assay for multiple HIV transcripts (read-through, TAR, elongated, spliced, etc.) which may yield insights into the nature of blocks to HIV transcription from the reservoir at baseline or in response to latency reversal agents. Inducible HIV cell-free RNA from culture supernatants can also be measured (Spivak et al., 2015). Furthermore, measurements of residual viremia from individuals on suppressive ART using ultrasensitive single-copy HIV RNA assays (SCA) may serve as a measurement of inducible persistent HIV, as this residual viremia may reflect persistent HIV that is expressed following T cell activation due to normal immune physiology. SCA have limits of detection of 1 copy/mL of plasma (or lower, depending on the volume of plasma used), and have been used to study ART treatment intensification and latency reversal in clinical studies (reviewed in Wang and Palmer, 2018).

Other RNA PCR approaches have been developed to estimate the frequency of RNA expressing reservoir cells. Limiting dilution single cell RNA PCR assays estimate the frequency of HIV cell-associated RNA expressing cells (Pasternak and Berkhout, 2018). A major limiting dilution assay is the Tat/rev induced limiting dilution assay, or TILDA (Procopio et al., 2015). By assaying multiply spliced HIV RNA, TILDA assay reduces reservoir frequency overestimation because multiply spliced HIV RNA is less likely to (but still may) have replication defects than DNA measurements (Procopio et al., 2015; Imamichi et al., 2016; Pollack et al., 2017). Another innovative approach for estimating the frequency of RNA-expressing cells is single cell ddPCR (Yucha et al., 2017). Of note, all of these assays require further cross-sectional and longitudinal validation, to determine if they can categorize individuals in a useful way, or if they have appropriate discriminatory power to register the effect of a reservoir depletion strategy over time.

### HIV RNA Sequencing

Limiting dilution sequencing assays have been developed, but are restricted by cost and sequence coverage. However, these assays are able to estimate the number of RNA copies per cell in addition to the frequency of RNA-expressing cells (Pasternak and Berkhout, 2018). Wiegand et al. (2017) developed the cell-associated HIV RNA and DNA single-genome sequencing (CARD-SGS) assay to estimate the frequency of HIV proviruses that express RNA at baseline. In addition, Das et al. (2018) recently reported a multi spliced RNA assay called EDITS (envelope detection by induced transcription-based sequencing) based on RT-nested PCR amplification of multiply spliced *env* RNA followed by NGS.

The sequencing of HIV RNA by SGS/NGS has been performed in several experimental contexts. SGS remains the gold standard even though it is expensive and time consuming. The use of NGS, however, is limited. The frequency of PCR errors, PCR bias, recombination and rearrangement that occurs during NGS library construction need to be carefully considered. Methods such as primer ID (Jabara et al., 2011; Zhou et al., 2015) and alternative adaptor attachment strategies (Boltz et al., 2016) can mitigate the errors associated with NGS library preparation.

### HIV RNA Staining Assays

*In situ* hybridization (ISH) of RNA at the single cell level was developed in the 1990s, and subsequently modified to simultaneously detect HIV RNA and host or virus RNA or protein (Wong et al., 1997a; Schacker et al., 2000; Baxter et al., 2018; Deleage et al., 2018). The development of these assays is reviewed elsewhere (Baxter et al., 2018). We highlight the recent development of next-generation HIV ISH assays, such as RNAscope and FISH-flow, that enable the detection of single HIV RNA positive cells with microscopy visualization (Estes et al., 2017; Zhang et al., 2018) or flow cytometry (Porichis et al., 2014; Martrus et al., 2016; Baxter et al., 2017; Grau-Exposito et al., 2017). Using these techniques, some inference about the levels (copies) of RNA per cell can be made based on staining intensities, in addition to the frequency of HIV RNA-expressing cells. A novel multiplex microscopy ISH approach allows for detections of viral DNA, RNA and protein. However, this has yet to be evaluated in cells from ART-suppressed participants (Puray-Chavez et al., 2017).

### HIV *gag* p24 Assays

Recent efforts in the cure field have developed assays to quantify baseline or inducible expression of viral proteins, in particular the p24 capsid protein in whole cells. The rationale behind this effort is that quantification of inducible viral protein reduces overestimation of the frequency of the replication-competent reservoir, as proviruses that can progress through transcription and translation are less likely to have major defects (Bruner et al., 2016). All assays that employ antibodies to p24 are limited by a false positive rate from non-specific antibody binding. The use of two p24 antibodies may, at least partially, overcome this limitation.

Graf et al. (2013) demonstrated that in ART-suppressed participants, *gag* protein positive cells could be detected at a frequency of ∼1 *gag*+ cell per million PBMCs. This finding supported the notion that at baseline, some persistent HIV is expressed at a protein level. However, this technique had a high false positive rate. To mitigate false positives, groups endeavored to detect *gag* positive cells in tandem with CD4 downregulation, suggestive of the presence of the Nef protein. O’Doherty and colleagues used a combination of Fiber-optic Array Scanning Technology (FAST) and automated digital microscopy to detect *gag* positive with concomitant CD4 downregulation, suggesting the presence of actively transcribed and translated viral proteins (DeMaster et al., 2015). While this technology improved the false positive rate, it is not yet widely used. Other groups focused on honing the specificity of p24 flow cytometry assays. The combination of *gag* staining with RNA flow (discussed above) proved useful in some trials but not others, likely due to RNA sequence heterogeneity (Baxter et al., 2016, 2017; Martrus et al., 2016; Grau-Exposito et al., 2017). Using a high number of RNA probes resulted in robust detection of *gag*+RNA+ cells, with the expected CD4 downregulation and enrichment in cell populations such as T_fh_ and PD1+ cells (Baxter et al., 2016, 2017, 2018).

More recent efforts have focused on using two *gag* protein probes in flow cytometry application to quantify inducible persistent HIV at the protein level. Chomont and colleagues recently published a flow cytometry protocol that utilizes two capsid antibodies for dual intracellular staining of p24 (Pardons et al., 2019). In ART-suppressed individuals, p24 double positive cells correlated with TILDA and QVOA measures. However, in this study, no p24 double positive cells were detected in ART-suppressed participants at baseline, in contrast to previous work using a single p24 antibody (Graf et al., 2013; Baxter et al., 2016). Notably, this novel technology might permit characterization of reservoir cells from ART-suppressed donors using standard flow cytometry surface markers and intracellular staining.

Another focus in quantification of viral reservoirs has utilized digital ELISA technology to quantify the very low levels of p24 produced from CD4 T cells after stimulation with a latency reversal agent or global T cell activator. This assay can be applied to cell lysates or culture supernatants and is able to detect sub-femtomolar levels of p24; however, the correlation of these measures with other assays of persistent HIV infection is not yet fully assessed (Wu et al., 2017).

Finally, high parameter mass cytometry techniques using heavy-metal tagged antibodies have been applied to *in vitro* studies of HIV infection to phenotype tonsillar CD4 T cells infected with a mass-cytometry compatible reporter virus (Cavrois et al., 2017). Assays to detect HIV p24 using mass cytometry are an area of active investigation and development.

## Outgrowth Assays

### HIV Outgrowth Assays

Significant effort has been deployed recently to develop new or improved assays to measure clinical interventions aimed at depleting the HIV replication-competent reservoir. While assays based on PCR of HIV DNA offer a streamlined method to quantitate persistent HIV infection, a significant fraction of persistently infected cells harbor defective proviruses that cannot be distinguished from replication-competent provirus by amplification of one genomic region (Eriksson et al., 2013; Ho et al., 2013; Bruner et al., 2016). Thus, approaches to eradicate HIV infection will likely need to incorporate assays that reflect depletion of replication-competent viruses to measure the efficacy of anti-latency therapeutics. The quantitative viral outgrowth assay (QVOA or simply VOA) is probably the most well-known assay used to quantitate replication-competent HIV.

During the early days of the HIV epidemic, co-culture assays of PBMC from HIV-infected individuals with Phytohaemagglutinin (PHA)-stimulated PBMC from uninfected donors were used to confirm the presence of replicating virus and provide an estimation of the viral burden within infected people (Castro et al., 1988; Jackson et al., 1988; Ho et al., 1989; Dimitrov et al., 1990). As it became evident that resting CD4 T cells were a major source of integrated proviral DNA during therapy (Chun et al., 1995), a derivative of these earlier co-culture assays was developed to quantify replication-competent HIV in purified CD4 T cells from HIV-infected individuals (Finzi et al., 1997; Wong et al., 1997b; Siliciano and Siliciano, 2005). Several detailed protocols for the traditional QVOA are available (Siliciano and Siliciano, 2005; Laird et al., 2016; Archin et al., 2017). Highly purified total or resting CD4+ T-cells are isolated from whole blood, leukapheresis product, or tissue-derived mononuclear cells for QVOA, and are stimulated with mitogen and co-cultured in a limiting dilution format with PHA-activated CD8-depleted PBMCs from HIV-seronegative individuals to exponentially expand the virus. Supernatant from co-culture assays are then assessed for the presence of p24 protein and Poisson statistics applied to estimate the frequency of resting cell infection that is reported as infectious unit per million CD4+ T cells (IUPM).

The QVOA is considered the gold standard for measuring replication-competent HIV and provides a definitive minimal estimate of reservoir size. However, the assay is costly and labor intensive, and given the low frequency of latently infected cells, often large numbers of cells are required to increase the limit of detection. The QVOA reports a minimal estimate of the frequency of latent but replication-competent proviruses, due to the presence of “non-induced” proviruses unresponsive to a single round of cell stimulation (Ho et al., 2013; Hosmane et al., 2017). Despite its limitations, the QVOA remains a reproducible and reliable method to measure the HIV latent reservoir and has been used to accurately assess the stability the reservoir over time. Using the QVOA, we measured resting CD4+ T-cell infection (RCI) in 37 HIV+ participants, on stable suppressive ART who donated resting CD4+ T cells via leukapheresis, over a period of 6 years and found that despite the long duration of ART, the half live of the reservoir in resting CD4+ T cells to be about 43 months, a value that is very close to that reported by Siliciano et al. (2003) a decade earlier, using an earlier version of the QVOA in a clinical cohort 10 years prior, on older ART (Crooks et al., 2015). This highlights (1) the stability of the replication-competent reservoir and (2) the reliability of the QVOA to probe reservoir dynamics.

Given the importance of the QVOA in assessing replication-competent HIV, many laboratories have introduced modifications to the original assay to streamline the assay and improve upon throughput, sensitivity and dynamic range (Avalos et al., 2016; Laird et al., 2016; Charlins et al., 2017; Fun et al., 2017; Metcalf Pate and Blankson, 2017; Sanyal et al., 2017; Massanella et al., 2018; Rosenbloom et al., 2019) (Table 2). Examples of modifications made to the standard QVOA to streamline and improve sensitivity involved replacing the uninfected donor PHA-blasts normally used in the co-culture to expand virus, with cell lines permissive for HIV infection such as the MOLT-4/CCR5, or using RT-PCR with primers specific for polyadenylated HIV-1 or *gag* RNA to detect virus released into culture supernatant (Laird et al., 2013; Massanella et al., 2018). In their mQVOA assay Massanella et al. (2018) also use the MOLT-4/CCR5 to propagate virus, but used immobilized CD3/CD28 beads to stimulate CD4 T cells and captured cell free RNA from the supernatant using magnetic beads before assaying HIV *gag* by PCR. In a recent cross-laboratory reservoir assay validation study (RAVEN), the above modification was reported to generate IUPM values that were 9.2 fold higher than traditional QVOA assays as RNA detection was thought to be more sensitive than p24 measurements (Rosenbloom et al., 2019). However, while PCR measurements as readout for the QVOA may provide more sensitivity, the potential that some of the RNA (even though released into the supernatant) detected could come from defective provirus, is not full length, and/or is not part of a viral particle should be taken into consideration (Pollack et al., 2017). RNA detection may also inherently be more prone to false positive detection due to low-level PCR product contamination.

**TABLE 2:** Overview of modified qVOA assays.

**Assay Name**	**Modification/Format**	**Readout**	**Reference(s)**
QVOA2	PHA+ sero-negative γ-irradiated PBMC stimulation of resting CD4+ T cells; MOLT4/CCR5 or SupTl-CCR5 as targets for virus expansion	PolyA RNA qPCR or p24 ELISA	Laird et al., 2013; Fun et al., 2017
TZA	CD3/CD28 stimulated CD4+ T cells are co-cultured with TZM-bl indicator cells	B-gal activity by chemiluminescence	Sanyal et al., 2017
dQVOA	Resting CD4+ T Cells are differentiated to effector memory phenotype before activating with PHA + seronegative γ-irradiated PBMC; no targets added	p24 ELISA	Wonderlich et al., 2019
mQVOA	Immobilized CD3/CD28 simulation of CD4+ T cells; MOLT4/CCR5 targets; cell free RNA in supernatant captured by magnetic beads	*gag* or *pol* RNA qPCR	Massanella et al., 2018; Rosenbloom et al., 2019
Murine VOA (mVOA)	CD4+ T cells or PBMC from aviremic donors engrafted into immunodeficient NSG mice, followed by administration of anti- CD3/CD28 and anti-CD8 antibodies	Plasma HIV RNA detection *(gag* primers) by qPCR	Metcalf Pate et al., 2015
Humanized mouse VOA (hmVOA)	CD3/CD28 stimulated resting CD4 T cells from aviremic donors are engrafted into humanized mice	Plasma HIV RNA detection (LTR primers) by qPCR	Charlins et al., 2017
MΦ-QVOA	Myeloid cells from SlV-infected macaques are stimulated with TNF-α; co-cultured with CEMX174 as targets	SIV *gag* RNA qPCR	Avalos et al., 2016

The introduction of ultra-sensitive p24 assay platforms have ushered in additional methods to quantitate latent HIV with improved sensitivity of antigen detection in culture supernatant (Descours et al., 2017; Passaes et al., 2017; Wu et al., 2017). We have developed a modified QVOA, the Digital ELISA Viral Outgrowth or DEVO assay that takes advantage of the recently developed SIMOA platform (Quanterix), capable of femtogram detection of HIV p24 protein in contrast to the picogram limitations of traditional ELISA. In this assay, 8–10 × 10^6^ purified resting CD4+ T cells from aviremic, ART-treated HIV+ participants are PHA stimulated in limiting dilution in a 96 well-format. With this assay we found that virus can be expanded using either the CD4 T cell input (i.e., addition of exogenous donor cells is not necessary), PHA blasts from an uninfected donor, or HIV permissive cell lines such as the MOLT4/CCR5. Furthermore, with the DEVO assay we obtained IUPM comparable or higher than the traditional QVOA at an earlier time point, thus reducing the overall length of the assay (Archin et al., 2018).

Finally, another modification of the QVOA reported recently involves differentiating the input memory CD4+ T cells into effector memory T cells before activation. On average, higher IUPM values were observed with the differentiated QVOA (dQVOA) compared to traditional QVOA assay methods (Wonderlich et al., 2019). This suggests that induction into the effector state prior to activation facilitates more efficient exit from latency (Kulpa et al., 2019).

## Reducing the Reservoir: Stability and Meaningful Reduction

For studies that aim to deplete the reservoir, it is critical to understand how specific reservoir assay measurements (HIV DNA, QVOA, etc.) change over time on suppressive therapy without any cure intervention. Studies of reservoir stability to date have employed both bulk and intact DNA PCR (PBMCs, peripheral CD4 T cells) (Besson et al., 2014; Laanani et al., 2015; Bachmann et al., 2019; Bruner et al., 2019) and viral outgrowth (peripheral CD4 T cells) (Siliciano et al., 2003; Crooks et al., 2015). Generally, in peripheral blood CD4 T cells, QVOA and IPDA reservoir measurements have similar decay kinetics (t_1/2_ ∼44 months) (Bruner et al., 2019). Measures of bulk DNA or non-intact provirus are more variable in stability (Besson et al., 2014; Laanani et al., 2015; Bachmann et al., 2019; Bruner et al., 2019). Within tissues, little is known about reservoir stability because of the difficulties with accessing tissues, especially over multiple time points in human participants. Therefore, thorough understanding of the reservoir stability within tissue may depend primarily on animal models (Henrich et al., 2017).

In addition to reservoir stability, understanding inter-assay variability and the error associated with each measurement is essential to robustly assess potential reservoir reductions. For instance, evaluation of longitudinal QVOA data suggests that a sixfold decline in the frequency of resting CD4 T cell infection would be necessary to achieve 95% confidence that the intervention resulted in the reduction of the reservoir observed (Crooks et al., 2015). For other assays, however, the decline in persistent HIV frequency to achieve 95% confidence of a reservoir reduction is unknown, and further complicated by the presence of large frequencies of non-intact proviruses. The IPDA is a promising assay to assess reservoir reductions in a high-throughput manner; however, this must be tempered with concerns about interindividual variability for false positive non-intact proviruses and the need for further understanding of the stability and reproducibility of the IPDA over multiple time points and across diverse human cohorts (Bruner et al., 2019; Gaebler et al., 2019).

There is also rationale to conduct bulk measures of HIV provirus (i.e., DNA PCR of a conserved region of a viral gene) to assess the effect of reservoir reduction interventions on global levels of HIV DNA. Although >90% of HIV provirus is replication-defective, a significant portion of proviruses can express viral proteins that may contribute to chronic immune activation and sequelae (Hunt et al., 2016; Imamichi et al., 2016; Pollack et al., 2017). Therefore, information about the effect of interventions on total persistent HIV frequency may be useful. At the same time, however, defective provirus may be more resistant to latency reversal and immune clearance interventions because of lower expression of viral RNA and/or protein. If this is the case, reservoir assays that measure intact proviruses, viral RNA/proteins, or replication-competent HIV may have a higher sensitivity to potential reservoir reductions.

For experimental medicine trials, we propose a framework where the more costly and labor intensive QVOA-based measures are employed at study beginning and as primary end points, with more intensive readouts of genetically intact DNA, bulk DNA, and/or other reservoir measurement occurring both at the start and end, as well as throughout the trial. Our reasoning behind this recommendation is that QVOA provides a direct measure of replication-competent virus that could re-establish an infection, whereas DNA and other assays all have concerns about detection of replication-deficient virus. Moreover, for QVOA, there is a defined magnitude of decline (sixfold) in reservoir frequency necessary to attribute a reservoir reduction to a specific intervention (Crooks et al., 2015), whereas for other assays this magnitude of reduction is unclear. In addition, for potential cures, it will be essential to show by QVOA-based methods over multiple time points the absence of replication-competent virus recovery. The engraftment of CD4+ T cells from individuals who are negative by QVOA into murine viral outgrowth models (mVOA or hmVOA, Table 2) could be used as additional metrics to ascertain the absence or reduction of replication competent virus (Metcalf Pate and Blankson, 2017).

At the same time, however, standard QVOA based measurements only provide a definitive minimal estimate of reservoir frequency, likely because of the presence of non-induced proviruses (Ho et al., 2013). Concomitant use of the IPDA overcomes this limitation because measurement of genetically intact provirus is not dependent on the transcriptional status of the cell, and may provide a maximal estimate of replication-competent provirus. Further, other reservoir measurements such as the IPDA or other assays described herein may afford a greater dynamic range and sensitivity to reservoir reductions, though this remains to be proven. As there is growing evidence for the clonality of the HIV reservoir, novel full length sequencing approaches with integration site analysis will permit the assessment of the impact of interventions on the clonality of the reservoir. Taken together, the combination of primary endpoint QVOA-based reservoir measures with more intensive, higher throughput evaluation of the reservoir using the IPDA, bulk DNA, inducible reservoir measurement(s), and/or sequencing approaches will enable a thorough evaluation of the impact of any intervention on persistent HIV in cure research trials.

## Summary

Assays to measure persistent HIV infection are emerging quickly. Key issues for the cure field with regard to reservoir assay development include (1) optimization of viral outgrowth assays for reproducibility, sensitivity, and dynamic range, (2) further understanding of interindividual variability in false positive intact proviruses and stability over time for the IPDA, (3) additional development of full-length/integration site sequencing approaches to understand proviral dynamics during therapy or potential interventions, and (4) advancement of DNA/RNA/p24 single and/or multiplex detection methods to better understand the dynamics and mechanisms of latency reversal with different agents.

## Author Contributions

SF, CC, DM, and NA conceptualized and outlined the manuscript. SF, CC, and NA wrote the first draft of the manuscript. All authors edited and approved the final version.

## Conflict of Interest

The authors declare that the research was conducted in the absence of any commercial or financial relationships that could be construed as a potential conflict of interest.
